# Differential Influences of the Aryl Hydrocarbon Receptor on Th17 Mediated Responses *in vitro* and *in vivo*

**DOI:** 10.1371/journal.pone.0079819

**Published:** 2013-11-14

**Authors:** João H. Duarte, Paola Di Meglio, Keiji Hirota, Helena Ahlfors, Brigitta Stockinger

**Affiliations:** Division of Molecular Immunology, MRC National Institute for Medical Research, London, United Kingdom; Escola Paulista de Medicina – UNIFESP, Brazil

## Abstract

The aryl hydrocarbon receptor (AhR) has been attributed with anti-inflammatory effects in the development of pathological immune responses leading to experimental autoimmune encephalomyelitis (EAE) via the induction of regulatory T cells. In agreement with previously published findings, we find that TCDD administration confers protection from EAE, however, this immuno-modulatory effect was not the consequence of *de novo* Treg generation, but the inhibition of Th17 cell differentiation. Systemic application of FICZ at the time of immunization also reduced EAE pathology albeit to a lesser degree than TCDD. *In vitro* Th17 differentiation in the presence of AhR agonists, including TCDD, promoted IL-17 and IL-22 expression, but did not induce Treg differentiation. AhR affinity influenced the amounts of IL-17 and IL-22 protein that was secreted by Th17 cells, but did not seem to affect susceptibility to EAE *in vivo*. Making use of conditional AhR-deficient mice, we show that the anti-inflammatory effect of TCDD depends on AhR activation in both T cells and dendritic cells, further emphasising the ability of TCDD to interfere with T effector cell differentiation *in vivo*. The dichotomy between the *in vivo* and *in vitro* effects of AhR reveals the complexity of the AhR pathway, which has the capacity of affecting different AhR-expressing cell types involved in mounting immune responses, thus participating in defining their outcome.

## Introduction

CD4^+^ T lymphocytes expressing interleukin (IL)-17 (Th17 cells) constitute a distinct subset of effector T cells with a specific transcriptional program, defined by the lineage determining factors RORγt and RORα[1], [2]. Despite their crucial contribution to protection against extracellular pathogens [3], Th17 cells have been implicated in the immunopathology of a range of inflammatory diseases such as psoriasis [4], rheumatoid arthritis [5] and multiple sclerosis [6], [7].

The ligand-dependent transcription factor aryl hydrocarbon receptor (AhR), best known for mediating the transcriptional response to xenobiotics such as 2,3,7,8-tetrachlorodibenzo-p-dioxin (TCDD) [8], was shown to enhance the Th17 developmental program [9] and to induce the expression of the cytokine IL-22 in Th17 cells [10]. Th17 cells play a crucial role in the pathogenesis of experimental autoimmune encephalomyelitis (EAE), a murine model of multiple sclerosis (MS), where invasion of the central nervous system (CNS) by myelin-specific Th17 cells leads to neuronal degeneration and progressive limb paralysis [11]. Autoimmune diseases such as MS are multifactorial and, in addition to genetic factors, environmental factors influence the initiation and progression of disease. Thus, the involvement of AhR agonists in shaping the course of autoimmune pathology could constitute a link to environmental factors that influence autoimmune disease. While AhR-deficient mice developed a much milder form of EAE with many mice protected from onset of disease altogether [10], the application of AhR agonists caused differential effects. The local administration of the tryptophan metabolite 6-formylindolo(3,2-b)carbazole (FICZ), an endogenous AhR agonist [12], exacerbated disease [10], while systemic administration of TCDD had the same ameliorating effect on disease progression as AhR-deficiency [9]. This led to the suggestion that AhR exerts its effects on immune responses in a ligand-dependent manner. AhR activation has been reported to have an overall anti-inflammatory effect, which was linked to induction of regulatory T (Treg) cells [9], [13], promotion of the differentiation of IL-10-expressing Tr1 cells [14], [15], and induction of a tolerogenic phenotype in dendritic cells (DC) [16]–[18].

Here we show that AhR ligands such as TCDD and FICZ both upregulate the Th17 program *in vitro* with the magnitude of response depending on AhR affinity. Their effects *in vivo* depend on timing and mode of application, suggesting that the mode of action of these two AhR ligands *in vivo* is more likely to be shaped by their differential susceptibility to metabolic feedback control. TCDD is the most stable of all xenobiotic AhR ligands [19], whereas FICZ is rapidly metabolised by AhR induced cytochrome P450 enzymes [20], thus causing only transient AhR signalling. Furthermore, we show that limiting AhR deficiency to defined haematopoietic cell types results in partial alleviation of the suppressive effect of TCDD on EAE development.

## Materials and Methods

### Ethics Statement

All animal experiments were approved by the local Ethical Review panel at NIMR in accordance with the Institutional Committees on Animal Welfare of the UK Home

Office (the Home Office Animals Scientific Procedures Act, 1986)

### Mice

C57Bl/6, Rag1.Cre [21], CD11c.Cre (Jackson stock 008068 B6.Cg-Tg(^Itgax-cre^)1-1Reiz/J [22], AhR^fl/fl^ (Jackson Stock 006203 B6.129(FVB-Ahr^tm3.1Bra/J^), AhR^−/−^ (B6 BRA AHRKO) [23] and Foxp3eGFP reporter mice [24] were bred in the NIMR animal facility under specified pathogen free conditions.

### EAE induction

Mice were injected at the base of tail with 100 µl emulsion of IFA containing 250 µg MOG peptide fragment 35–55 and 250 µg *Mycobacterium tuberculosis* strain H37Ra, followed by 200ng *Bordetella pertussis* (Calbiochem) i.p. on the day of immunization and two days later. Clinical assessment of EAE was performed daily and clinical scores were assessed according to the following criteria: 0  =  unaffected, 1  =  flaccid tail, 2  =  impaired righting reflex and/or gait, 3  =  partial hind limb paralysis, 4  =  total hind limb paralysis, 5  =  total hind limb paralysis with partial fore limb paralysis. 1 µg/mouse TCDD or 200 µg/mouse FICZ (or olive oil as vehicle control) were given as a single i.p. dose of as described [9] either on the day of MOG/CFA immunization or 5–7 days later.

### Analysis of draining lymph nodes and spinal cord infiltrating cells

At the time points depicted, single cell suspensions were obtained from paraaortic lymph nodes or from the spinal cord by mechanical dissociation. Anti-αβTCR (H57-597), anti-CD4 (GK1.5), anti-CD25 (PC61.5), anti-CD44 (IM7), anti-Foxp3 (FJK-16s), anti-IFN-γ (XMG1.2), and anti-IL-17A (TC11-18H10.1) were obtained from eBioSciences. Anti-IL-1R1 (JAMA-147) and anti-CCR6 (140706) were purchased from Biolegend. Anti-IL-22 (MH22B2) was purified from culture supernatant of the hybridoma and labelled in our laboratory [10]. For intracellular cytokine staining cells were stimulated for 4 h with PdBU (500 ng/ml) and ionomycin (500 ng/ml) in the presence of brefeldin A (1 µg/ml) and Fc block (BD), while simultaneously stained for surface markers (CD4, TCRβ), then fixed with 3.8% PFA, permeabilized with 0.1% NP-40 and stained for IL-17A and IFN-γ (Biolegend). Intranuclear staining for Foxp3 was performed using the Foxp3 Staining Kit (eBioscience) according to the manufacturer’s instructions. Cells were acquired on a FACSCanto II (BD) and data analysis was performed using FlowJo (TreeStar) software.

### *In vitro* T cell differentiation and intracellular staining

Naïve CD4 T cells (CD4^+^, CD25^−^, CD44^−^) were isolated by FACS sorting using a MoFlo XDP (Beckman Coulter) and cultured in Iscove’s modified Dulbecco medium (IMDM, Sigma) supplemented with 2×10^−3^M L-glutamine, 100 U/ml penicillin, 100 µg/ml streptomycin, 5×10^−5^ M β-mercaptoethanol and 5% fetal calf serum. Th17 and Treg cells were differentiated on plates coated with 2 µg/ml anti-CD3 + 5 µg/ml anti-CD28, with a cytokine cocktail of 50 ng/ml IL-6, 1 ng/ml TGFβand1d ng/ml IL-1 (Th17) and 5 ng/ml TGFβ (Treg). Intracellular cytokines were measured as described above on day 4 after initiation of cultures. AhR ligands were tested on T cells *in vitro* in a range of concentrations to determine maximum effect without toxicity, and used at optimal concentrations in Th17 or Treg differentiation assays: TCDD was used at 100 nM and FICZ, ITE, 3MC and β-NF at 0.5 µM. For co-culture experiments, bone marrow derived DC (BMDC) were obtained by stimulating bone marrow cells with recombinant GM-CSF for 7 days and cultured with naïve T cells at a ration of 5:1 (T cells to DC). For proliferation analysis of Th17 cultures, T cells were stained with Cell Trace Violet (Invitrogen) for 20mins prior to culture; division index (average number of cell divisions each cell has undergone), replication index (fold-expansion of dividing cells) and percentage of divided cells were calculated using Proliferation platform with FlowJo software (Treestar).

### Determination of cytokines levels

IL-17A, IL-22 and IL-10 cytokine levels in cell supernatants were assayed using the Milliplex MAP Mouse Th17 Magnetic Bead Panel (Merck Millipore) according to the manufactures’ instructions, and acquired on a Bio-Plex 200 flow-based sorting and detection analyser (Bio Rad).

### RNA extraction and quantitative RT-PCR (qRT-PCR)

Total RNA was obtained using TRIzol (Life Technologies) according to the manufacturers’ instructions and reverse transcribed into cDNA. *ahr*, *foxp3* and *il1r1*

mRNA expression was assessed by real-time quantitative PCR using Taqman assays (Life Technologies) according to the manufacturers’ instructions. For each sample, mRNA abundance was normalized to the amount of mouse *hprt*. Data analysis was performed using the ΔCt method: results are expressed either as fold change or as relative mRNA levels.

### Statistical Analysis

Statistical analysis was performed using Prism version 5.0 (GraphPad Software). For EAE clinical score comparisons, values are expressed as the mean ± S.E.M. of n animals and data shown are representative of at least 2 independent experiments. Comparisons were calculated by two-way ANOVA test, followed by Dunnet post-test for multiple comparisons. Cell numbers and frequencies were compared by upaired t test or one-way ANOVA followed by Dunnet post-test for multiple comparisons. The level of statistically significant difference was defined as p ≤ 0.05.

## Results

### TCDD inhibits the establishment and progression of experimental autoimmune encephalomyelitis

**Figure 1:**
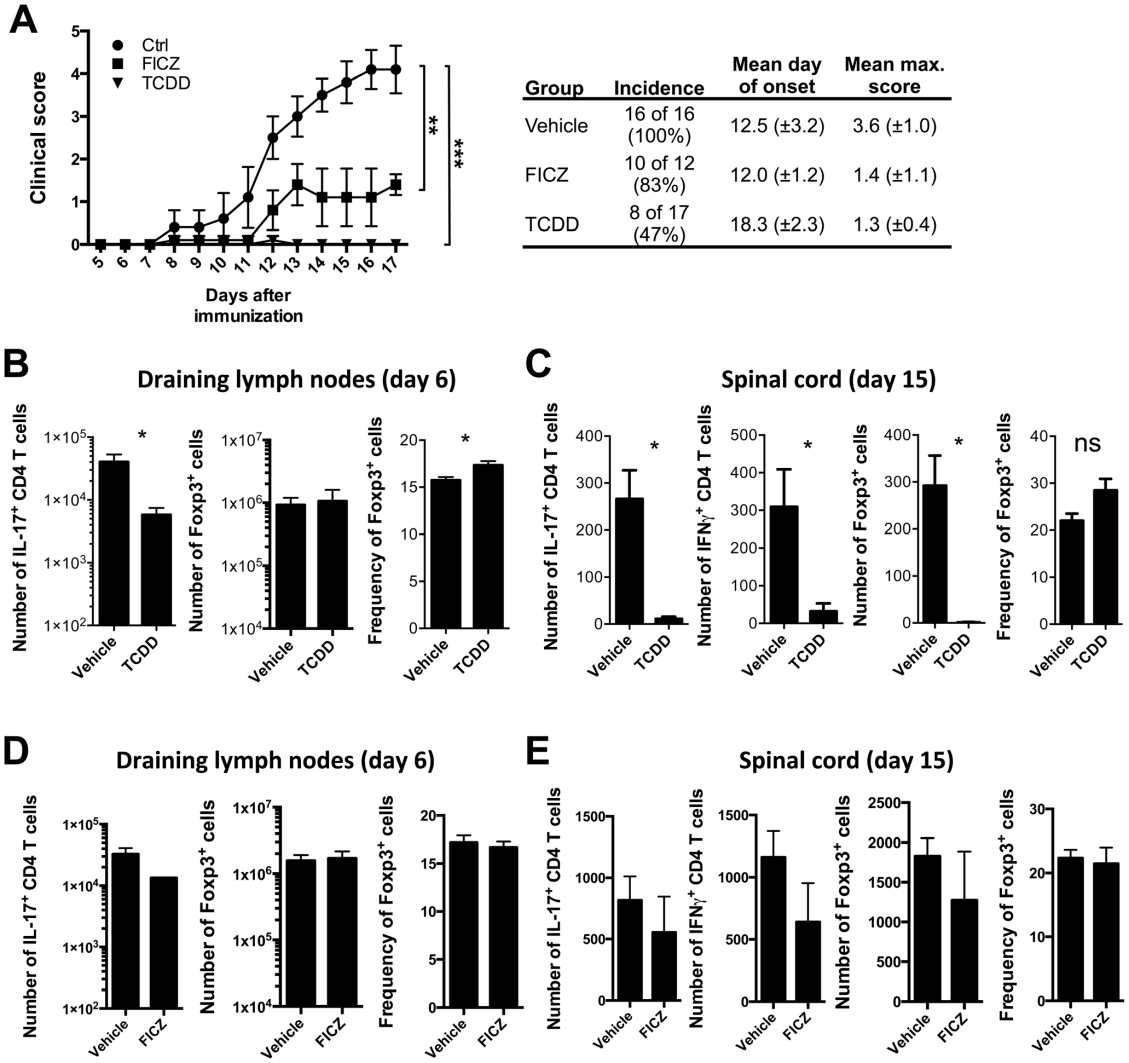
TCDD and FICZ administration inhibit EAE establishment and dampen T cell expansion and infiltration of the central nervous system. **A**) Clinical scores of mice immunized with MOG/CFA which received either vehicle (filled circles), TCDD (inverted triangles) or FICZ (filled squares) i.p. at the time of immunization; table showing incidence, mean day of onset and mean maximum score of disease. Data shown as mean ± SEM are representative of 2 independent experiments, ***p<0.001, **p<0.01 in two-way ANOVA followed by Dunnet post-test. **B**) Number of IL-17A^+^, and number and frequency of Foxp3^+^ T cells recovered from draining lymph nodes on day 6 post immunization in mice that received TCDD. Data shown as mean ± SEM are representative of 2 independent experiments. *p<0.05 in Student’s T test. **C**) Number of IL-17A^+^, IFN-γ^+^ and Foxp3^+^ T cells, and frequency of Foxp3^+^ T cells recovered from the spinal cord on day 15 post immunization in mice that received TCDD. Data shown as mean ± SEM are representative of 2 independent experiments. *p<0.05 in Student’s T test. **D**) Number of IL-17A^+^, and number and frequency of Foxp3^+^ T cells recovered from draining lymph nodes on day 6 post immunization in the presence of FICZ. **E**) Number of IL-17A^+^, IFN-γ^+^ and Foxp3^+^ T cells, and frequency of Foxp3^+^ T cells recovered from the spinal cord on day 15 post immunization in mice that received FICZ. Data shown as mean ± SEM are representative of 2 independent experiments.

The mouse model experimental EAE recapitulates some aspects of the progressive paralysis observed in MS patients. Following administration of Myelin Oligodendrocyte Glycoprotein Peptide (MOG) peptide emulsified in complete Freund’s adjuvant, myelin-reactive Th17 cells will undergo activation in lymph nodes and cross the blood-brain barrier, initiating an inflammatory cascade in the central nervous system which will lead to neuronal degeneration and gradual limb paralysis. In agreement with published data [9], administration of TCDD at the time of immunization systemically reduced disease incidence from 100% to 47%, and decreased the severity of disease in mice that show clinical manifestations of EAE (**Fig. 1A**). We and others have previously shown that local administration of FICZ (combined in the emulsion with antigen and CFA) exacerbates EAE [9], [10]. However, systemic administration of FICZ by intraperitoneal injection resulted in partial inhibition of EAE midway to that seen following administration of TCDD (**Fig. 1A**). To better characterize the clinical phenotype observed in immunized mice following systemic AhR activation, we assessed the number of T cells recovered from draining lymph nodes and spinal cord. Mice that had received TCDD showed significantly decreased numbers of Th17 cells in draining lymph nodes on day 6 after immunization, whereas at this time point there was no change in the number of Treg, albeit their frequency was slightly increased as reported previously [9] (**Fig. 1B**). In the spinal cord of TCDD-treated immunized mice the overall number of all infiltrating T cells, including Treg was dramatically decreased on day 15 after immunization (**Fig. 1C**). Following EAE induction in the presence of i.p.-administered FICZ, there were no significant changes visible in the draining lymph nodes (**Fig. 1D**), whereas the spinal cord showed an intermediate phenotype with respect to the vehicle control (**Fig. 1E**).

These observations imply that chronic activation of AhR prevents Th17 mediated immune inflammation in the central nervous system. Thus, the consequences of AhR activation during Th17-mediated immune responses appear to be dependent on the route of administration rather than the type of ligand.

### Immunosuppressive effect of TCDD depends on the timing of administration

**Figure 2:**
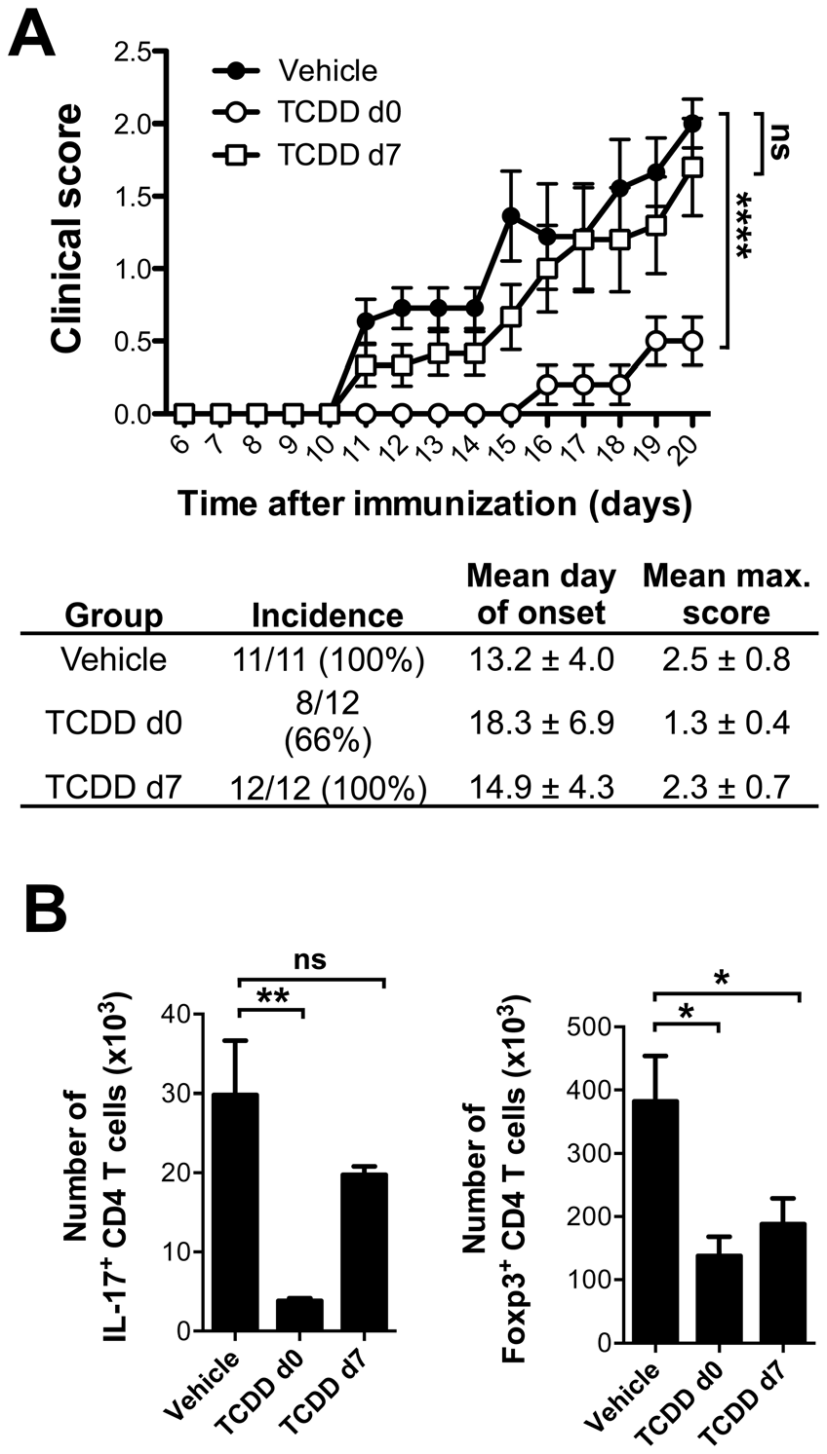
Administration of TCDD after initiation of the immune response does not result in immunosuppression. **A**) Clinical scores of mice immunized with MOG/CFA which received either vehicle (filled circles) or TCDD i.p. at the time of immunization (open circles) or 7 days after immunization (open squares). Table showing incidence, mean day of onset and mean maximum score of disease. Data shown as mean ± SEM are representative of 2 experiments, **** p<0.0001, two-way ANOVA followed by Dunnet post-test. **B**) Number of Foxp3^+^ and of IL-17A^+^ T cells recovered at day 9 after immunization from draining lymph nodes of immunized mice who received vehicle, TCDD on day 0 or TCDD on day 7 after immunization; n = 4. Data shown as mean ± SEM are representative of 2 experiments, *p<0.05, **p<0.01 in one-way ANOVA followed by Dunnet post-test.

As shown above, administration of TCDD simultaneously with the immunogen inhibited EAE development, and reduced the proportion and absolute numbers of IL-17 as well as IFN-γ-secreting CD4 T cells. In contrast, delaying administration of TCDD to 5 (data not shown) or 7 days after immunization allowed normal EAE development similarly to what was observed in the control group that did not receive TCDD (**Fig. 2A**). The number of Th17 cells recovered from the draining lymph nodes at day 9 after immunization in delayed TCDD administration was similar to that seen in vehicle immunized mice, and in strong contrast to the reduced numbers seen when TCDD was given concomitant with the immunization (**Fig. 2B**).

Whereas the number of Treg on day 6 after immunization was not altered with TCDD administration (**Fig. 1B**), when analysed on day 9 post immunization Treg numbers (but not percentages, data not shown) were partially reduced when TCDD was given either simultaneously with the immunization or 7 days after immunization (**Fig. 2B**). This suggests that AhR activation affects an early step in Th17 cell differentiation, which is no longer crucial once T cells are activated.

### AhR activation by TCDD *in vitro* promotes Th17 differentiation but not iTreg generation

**Figure 3:**
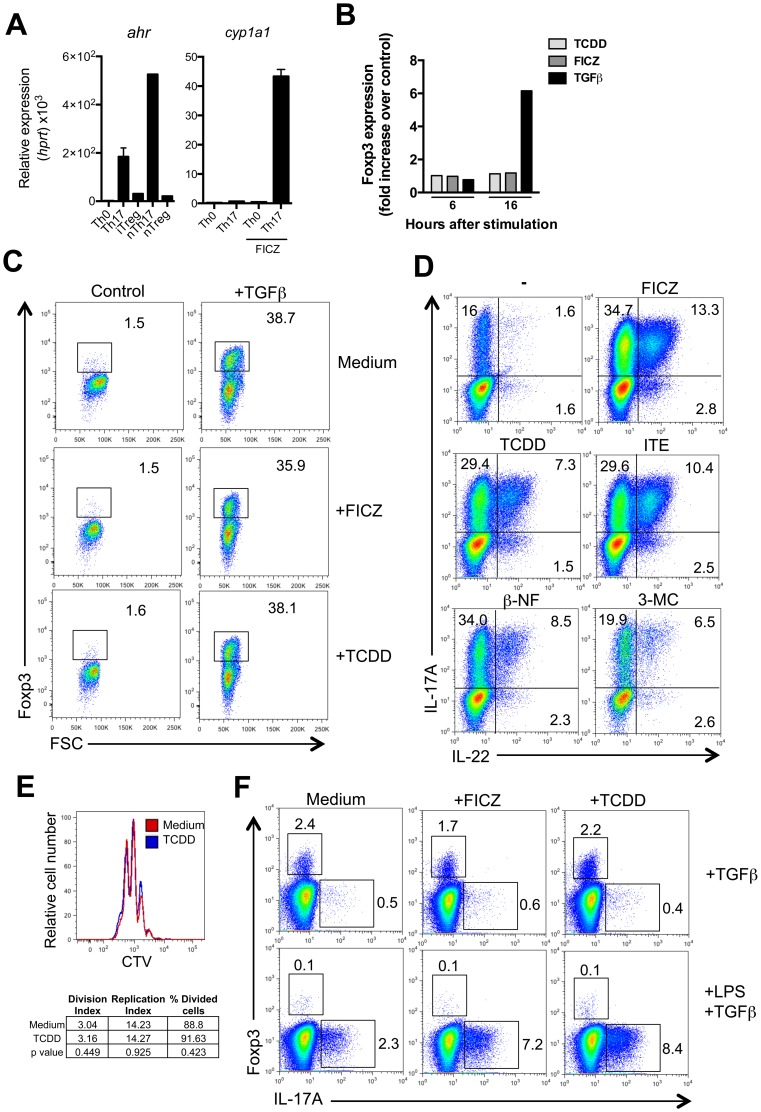
AhR activation by TCDD and FICZ *in vitro* promotes Th17 but not Foxp3^+^ Treg cells. **A**) Left panel: *ahr* expression from *in vitro* differentiated Th0, Th17 and Treg cells, and *ex vivo* sorted natural Th17 (nTh17) and Treg cells (nTreg) Data shown as mean ± SEM are representative of 2 experiments; n = 2–4. Right panel: *cyp1a1* expression from *in vitro* differentiated Th0 or Th17 in the presence or absence of FICZ. Data shown as mean ± SEM are representative of 2 experiments; n = 3. **B**) Fold increase of *foxp3* expression in cultured naïve T cells activated in the presence of TCDD, FICZ or TGFβ over control conditions without mediators at the indicated timepoint. **C**) Representative FACS dot plots showing Foxp3 staining of sorted naïve T cells activated in the presence of indicated ligands and cytokines. **D**) Representative FACS dot plots showing IL-17A and IL-22 staining in naïve T cell activated in the presence of IL-6 and TGFβ, plus the depicted AhR ligands. **E**) Representative histogram showing proliferation of *in vitro* differentiated Th17 cells (gated on IL-17^+^ cells) in the presence or absence of TCDD by CTV dilution; table shows the average division index (average number of cell divisions each cell has undergone), replication index (fold-expansion of dividing cells) and percentage of divided cells for each group, and the corresponding p value from Student’s T test analysis. **F**) Representative FACS dot plots showing IL-17A and Foxp3 staining in naïve T cell co-cultured with bone marrow derived DC in the presence of the indicated cytokines and ligands. Data are representative of 2 independent experiments.

It has been suggested previously [9], [13], [25] that TCDD induces Treg, whereas the endogenous AhR ligand FICZ promotes (but does not induce) Th17 differentiation. A prerequisite for an influence of AhR on differentiation of Treg is that it is expressed prior to differentiation. Expression of *ahr* measured by qPCR was not detectable in non-polarised activated T cells, whereas it was high in *in vitro* differentiated Th17 cells and in *ex vivo* isolated Th17 cells, but an order of magnitude lower in Treg, whether *in vitro* generated iTreg or *ex vivo* isolated nTreg. *Cyp1a1* expression was only detectable upon AhR stimulation under Th17 cell conditions as previously documented [10], [26] (**Fig. 3A** **)**. Previous studies also detected AhR expression in Tr1 cells (15). Upon culture of FACS sorted GFP-ve naïve CD4 T cells from Foxp3 reporter mice in the presence of TCDD and/or TGFβ there was no *foxp3* induction above background with TCDD, either measured by expression of *foxp3* in qPCR (**Fig. 3B**) or by FACS analysis for Foxp3-GFP reporting (**Fig. 3C**). In cultures with TGFβ-induced *foxp3,* addition of TCDD did not increase TGFβ-mediated induction of *foxp3*, making it unlikely that there is a major AhR mediated effect on iTreg generation. This is in concordance with our previous demonstration that naïve CD4 T cells do not express AhR [10]. In contrast, we observed that Th17 cell differentiation and the induction of IL-22 *in vitro* was substantially increased in the presence of a wide range of AhR ligands, including TCDD (**Fig. 3D**), showing that AhR activation has a ligand independent effect on Th17 promotion and IL-22 induction *in vitro*. There was no influence of TCDD on cell division during Th17 cell culture *in vitro* (**Fig. 3E**). Furthermore, Th17 cell differentiation in the presence of antigen presenting cells also showed similar enhancement by TCDD and FICZ, whereas there was no influence on Foxp3 induction under conditions that allow Treg differentiation (TGFβ without LPS) (**Fig. 3F**).

### AhR controls IL-1 receptor type 1 on Th17 cells

**Figure 4:**
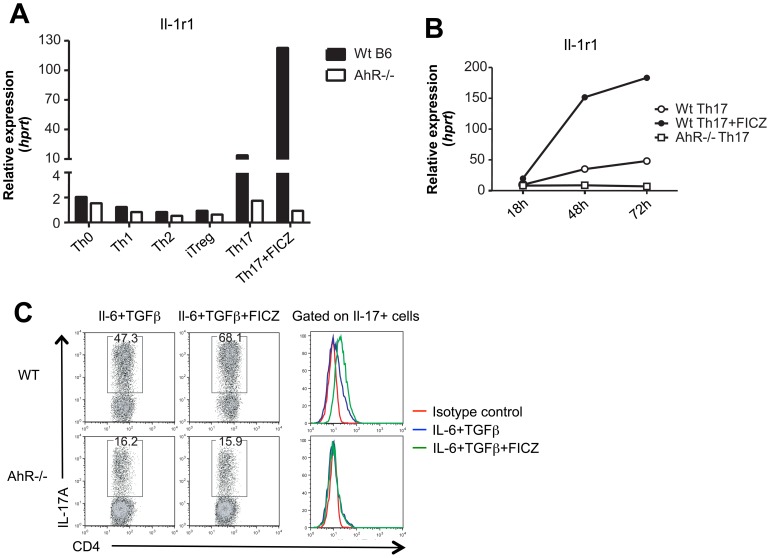
AhR positively controls IL-1 receptor type 1 on Th17 cells. **A**) Naïve CD4^+^ T cells from WT B6 (black bars) and AhR^−/−^ mice (white bars) were differentiated into Th0, Th1, Th2, iTreg, and Th17 cells for 3 days and the relative gene expression of Il-1r1 was determined by quantitative RT-PCR. **B**) Il-1r1 expression of WT Th17 cells cultured with (filled circles) or without FICZ (open circles) and AhR^−/−^ Th17 cells (open squares) was assessed at 18, 24, and 48h after the onset of Th17 differentiation. **C**) Th17 cells were induced from naïve WT CD4^+^ T or AhR^−/−^ naïve CD4^+^ T cells for 3 days and the expression level of surface IL-1R1 was analyzed gated on IL-17-producing cells. Data are representative of at least two independent experiments.

In a search for molecules controlled by AhR activation in Th17 cells, we had performed microarray analyses between WT and AhR-deficient Th17 cells in the presence of FICZ (data not shown). Amongst downstream targets upregulated by AhR activation such as IL-22, CYP1A1, CYP1B1 and the AhR repressor (AhRR), we identified the IL-1 receptor type 1 gene (*Il1r1*) as overexpressed in FICZ stimulated WT Th17 cells. Expression of the *Il1r1* is highly important for the effector function of Th17 cells as I*l1r1*-deficient animals fail to develop functional Th17 cell responses and consequently do not succumb to pathology in EAE [27], [28]. In order to validate the specific expression of *Il1r1*, Th0, Th1, Th2, iTreg, and Th17 cells were differentiated from naïve WT or AhR-deficient CD4 T cells and *Il1r1* mRNA expression was assessed by quantitative RT-PCR. *Il1r1* mRNA expression was barely detectable in CD4 T cell subsets other than WT Th17 cells, which robustly increased *Il1r1* in the presence of FICZ (**Fig. 4A**), whereas AhR-deficient Th17 cells showed impaired *Il1r1* mRNA expression. *Il1r1* mRNA levels increased during Th17 differentiation (**Fig. 4B**). Co-staining of surface IL-1R1 and intracellular IL-17 confirmed the reduced expression of IL-1R1 on AhR-deficient Th17 cells. Activation of AhR increased Th17 polarization and IL-1R1 expression in WT, but not AhR-deficient CD4 T cells (**Fig. 4C**).

### Similar Th17 differentiation in T cells bearing different AhR isoforms and stimulated with different AhR ligands

**Figure 5:**
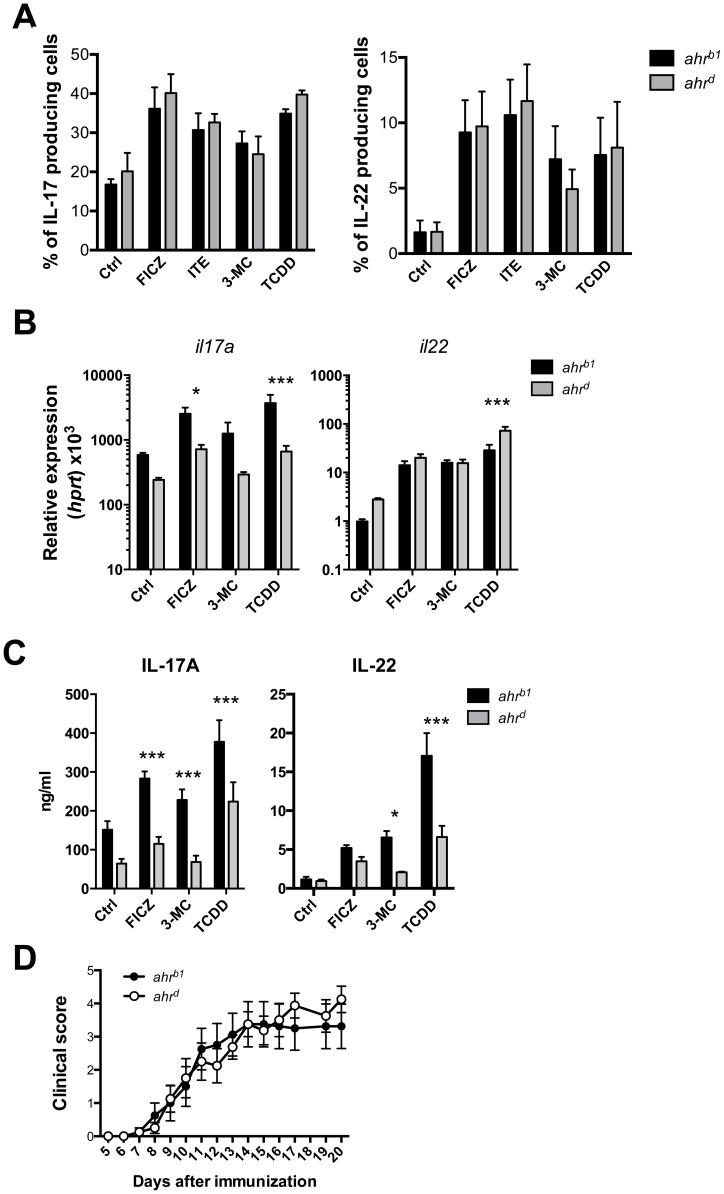
*ahr^b1^* and *ahr^d^* carrying mice show similar *in vitro* Th17 differentiation and *in vivo* disease kinetics following MOG-CFA immunization. **A**) Frequency of IL-17A (left panel) and IL-22 (right panel) -expressing cells following culture of naïve CD4^+^ T cells in Th17-promoting conditions in the presence of different AhR ligands for 4 days; cells bear either the *ahr^b1^* (black bars) or the *ahr^d^* (grey bars) allele for AhR. n = 3–4, data shown as mean ± SEM are representative of 2 independent experiments. **B**) mRNA expression of *il17a* (left panel) and *il22* (right panel) in Th17 cells cultured in the presence of different AhR ligands for 4 days; cells bear either the *ahr^b1^* (black bars) or the *ahr^d^* (grey bars) allele for AhR. n = 3–4, data shown as mean ± SEM are representative of 2 independent experiments. *** p<0.001 in a two-way ANOVA test. **C**) Quantification of IL-17A (left panel) and IL-22 (right panel) protein levels in supernatant obtained from Th17 cells cultured in the presence of different AhR ligands for 4 days; cells bear either the *ahr^b1^* (black bars) or the *ahr^d^* (grey bars) allele for AhR. n = 3–5, data shown as mean ± SEM are representative of 2 independent experiments. *** p<0.001 in a two-way ANOVA test. **D**) Clinical score of mice bearing either *ahr^b1^* (closed circles) or the *ahr^d^* (open circles) allele for AhR, immunized with CFA emulsified with MOG peptide. n = 8 mice per group.

A low affinity isoform of AhR (AhR^d^), which is the result of an Ala to Val change at residue 375 [29], was reported to have 10 fold lower affinity for TCDD [29] and we therefore compared the effect of different AhR ligands on differentiation of Th17 cells from B6 (AhR^b1^) and congenic B6 mice expressing AhR^d^. Culture of sorted naïve CD4 T cells under Th17 cell inducing conditions in the presence or absence of four different AhR agonists, in concentrations that were determined to induce the maximum polarization while averting any toxicity effects (data not shown), showed little difference between AhR^b1^ or AhR^d^ expressing strains with respect to induction of IL-17 and IL-22 (**Fig. 5A**). On the level of mRNA expression for Th17 cytokines there was a tendency of lower expression in AhR^d^ Th17 cells, which reached significance for TCDD and FICZ (**Fig. 5B**) and the effect was more pronounced on protein level where TCDD, FICZ and 3-MC all induced less IL-17 and IL-22 protein in Th17 cells from AhR^d^ compared with AhR^b^ mice (**Fig. 5C**). However, induction of EAE resulted in similar onset and level of pathology in AhR^d^ and AhR^b^ expressing mice (**Fig. 5D**).

### Deletion of AhR in either DCs or T/B cells causes reduction in EAE pathology following TCDD administration

**Figure 6:**
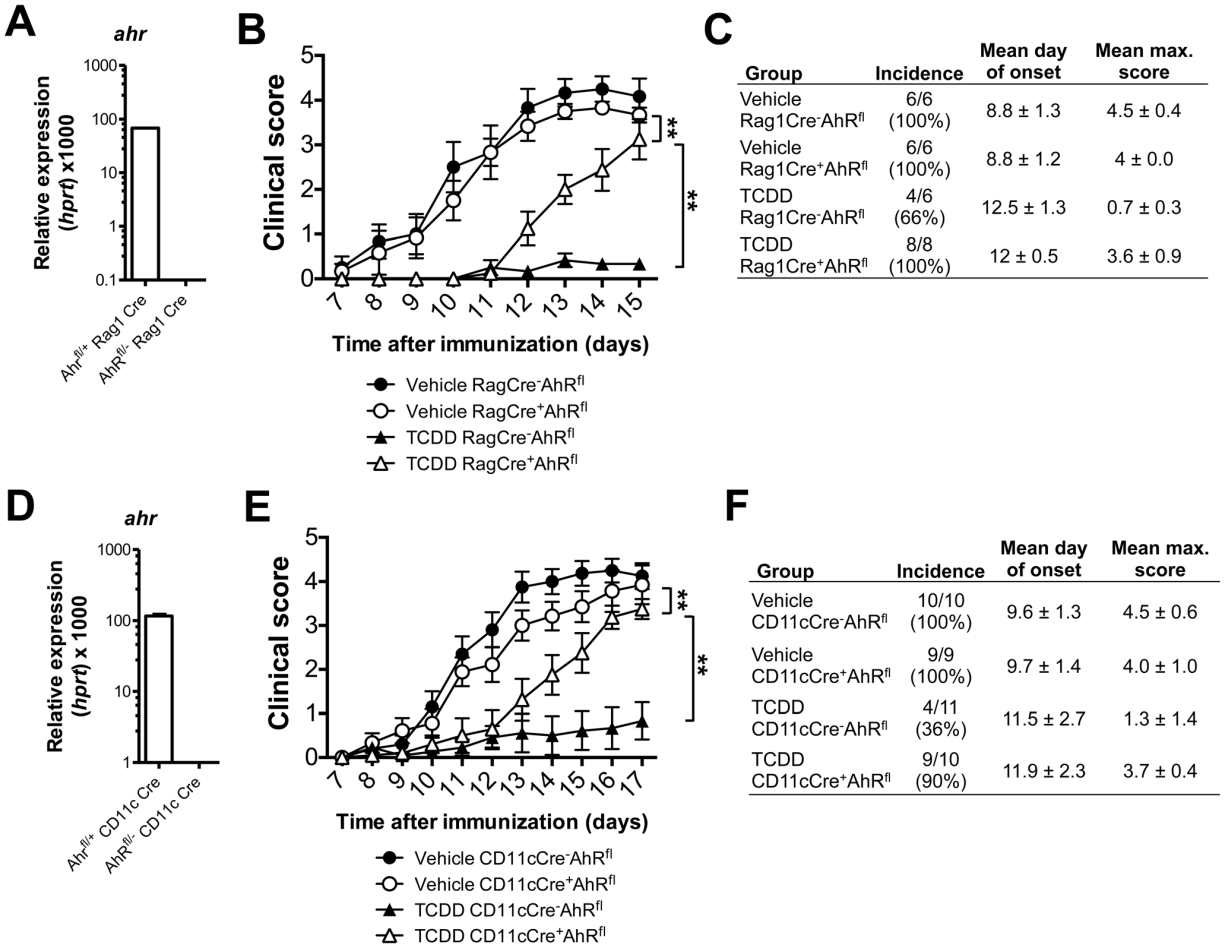
Restricting AhR deficiency to either T cells or dendritic cells partially abrogates TCDD mediated suppression of EAE. **A**) *ahr* mRNA expression in FACS sorted CD4 T cells from AhR^fl/+^ or AhR^fl/−^ Rag1.Cre mice. **B**) Clinical score of mice with Rag1-specific AhR-deletion (open circles, open triangles) or control mice immunized with MOG/CFA (filled circles, filled triangles) which received either vehicle (circles) or TCDD (triangles) i.p. at the time of immunization. n = 6–8, ** p<0.01 in two-way ANOVA followed by Dunnet post-test. **C**) Table showing incidence, mean day of onset and mean maximum score of disease for both experimental groups; n = 6-8, data shown as mean ± SEM. **D**) *ahr* expression in FACS sorted CD11c^+^ MHCII^+^ cells from AhR^fl/+^ or AhR^fl/−^ CD11c.Cre mice. **E**) Clinical score of mice with CD11c-specific AhR-deletion (open circles or open triangles) or control mice (filled circles, filled triangles) immunized with MOG/CFA which received either vehicle (circles) or TCDD (triangles) i.p. at the time of immunization. **F**) Table showing incidence, mean day of onset and mean maximum score of disease for both experimental groups; n = 9–11, data shown as mean ± SEM, * p<0.05 in two-way ANOVA followed by Dunnet post-test.

Next we sought to define the cell type targeted for the immunosuppressive effect of systemic AhR activation *in vivo*. For this we made use of conditional AhR-deficient mouse strains (AhR^fl/fl^) [30] and crossed them with mice expressing Cre recombinase under control of either the promoter for CD11c [22] or the promoter for recombination activating gene 1 (Rag1) [21], deleting AhR either in CD11c-expressing cell types (DCs, some macrophages and a proportion of NK cells) or in all T and B cells respectively (**Fig. 6A,D**). It should be noted that the AhR on the remaining cell types in such mice is of the low affinity AhR^d^ type, as the AhR^fl/fl^ mice were generated on an AhR^d^ background. We induced EAE in mice lacking AhR expressing in all Rag1-expressing cell types and controls (Rag1Cre^+^ AhR^fl/fl^ R26R eYFP and Rag1Cre^-^ AhR^fl/fl^ R26R eYFP). As shown in Fig. 5C, EAE onset and progression was similar in controls and experimental groups immunized with MOG/CFA and injected with vehicle (olive oil). However, the absence of AhR in T and B cells led to onset of EAE after treatment with TCDD with the same incidence as that seen in controls, but with the mean maximal scores reduced compared with control values (**Fig. 6B,C**). A similar effect was seen in mice with deletion of AhR in CD11c-expressing cells (**Fig. 6E,F**). Our findings indicate that EAE progression per se was not affected by the absence of AhR from T and B cells, or from dendritic cells/macrophages, but that the immunosuppressive effect of TCDD was partially lost when either of these cell types was AhR-deficient. These results suggest that the immunosuppressive effect of systemic TCDD administration is exerted through AhR expression on T cells as well as antigen presenting cells.

## Discussion

The AhR plays important physiological roles in many cells of the immune system, notably the Th17 subset of CD4 T cells [9], [10], [26], [31]. Most of the current literature on AhR effects in the immune system is focused on the consequences of exposure to the high affinity ligand TCDD. However, many recent studies of mice lacking AhR expression indicate that AhR activation affects important physiological functions in the absence of xenobiotic ligands [32]. There is a range of potential physiological ligands for AhR [33], [34] including diet-derived AhR ligands which strongly influence intestinal immune parameters [35]. A current controversy in the field is the apparent ligand-specific influence on the outcome of experimental models of autoimmunity such as EAE. The current paradigm is that TCDD promotes immune suppression, whereas endogenous ligands such as FICZ promote Th17 cell responses and thereby pathology [9]. However, in our view it seems more likely that the mode of application of a ligand rather than its nature defines the outcome. Thus, systemic administration of AhR ligands, affecting a multitude of tissues and cells types, appears to cause strong reduction of the concurrently induced immune response even in the case of FICZ, without the involvement of numerical changes or induction of Treg. In contrast, local injection of FICZ, which was incorporated into the antigen emulsion for induction of EAE seemed to more directly target and promote developing Th17 cells, thereby exacerbating pathology in EAE [10].

We decided to revisit the issue of ligand dependent effects of AhR looking at *in vitro* and *in vivo* Th17 cell responses in the presence of either TCDD or the endogenous ligand FICZ or other reported AhR ligands. It was clear that TCDD did not have differential effects compared to other AhR ligands during *in vitro* differentiation of Th17 cells and their induction of IL-22, suggesting that there is no intrinsically different mode of action distinguishing TCDD from other ligands. Affinity measurements for AhR polymorphisms have been conducted with respect to binding of TCDD in a hepatocyte cell line, and it is not clear at present whether other AhR ligands bind at the same location. This is an important issue as humans seem to express AhR of an affinity that is closer to that of AhR^d^ than AhR^b^ [36]. Affinity differences in AhR influenced the amounts of IL-17 and IL-22 protein that were produced by *in vitro* differentiated Th17 cells with significantly lower amounts secreted by cells from AhR^d^ mice. However, there was no difference in susceptibility to EAE between mice expressing AhR of low or high affinity. As this disease is strongly dependent on Th17 cells [11], [37], it appears that the number of Th17 cells in mice with low affinity AhR and the amounts of IL-17 protein produced are nevertheless sufficient to initiate disease.

It remains to be elucidated whether functional responses of other AhR expressing immune cell types are more or less affected by the affinity of AhR.

In contrast to its effect on *in vitro* Th17 cell differentiation, TCDD strongly suppressed Th17 cell development and the induction of EAE as described previously, a phenomenon that has been associated with the induction of Treg (reviewed in [38]). Similarly the suppression of EAE by another proposed endogenous ligand 2-(1'H-indole-3'-carbonyl)-thiazole-4-carboxylic acid methyl ester (ITE) was suggested to be due to the induction of Treg [17]. As all of the publications invoking induction of Treg by the AhR ligand TCDD demonstrate alterations in frequencies rather than absolute numbers, alternative interpretations for the apparent increase in Treg may apply. For instance, Treg cells are more resistant to apoptosis than conventional T cells [39], [40], surviving interventions that kill other T cells [41]–[43], which might result in alterations of relative frequencies. Given that AhR has long been known to interfere with regular cell cycle progression and that TCDD can induce cell cycle arrest [44], it is possible that T cell population dynamics are altered in the presence of TCDD with significant impact for the progression of immune responses. It should be noted that there was no obvious effect of TCDD on cell proliferation during Th17 cell differentiation *in vitro*. However, this does not necessarily rule out effects of TCDD on cell dynamics *in vivo*. We found no evidence for an induction of Foxp3 during exposure of naïve T cells to TCDD and TCDD did not influence the TGFβ mediated generation of iTreg *in vitro*. In addition we did not observe a numerical increase in Treg during induction of EAE despite an increase in frequency that has been reported previously. We therefore think it unlikely that the suppressive effect of TCDD manifests itself through the induction of Treg. This interpretation is furthermore supported by data in the literature that found no evidence of increased Treg in mice with constitutively active AhR [45].

Furthermore, it appears that while all AhR ligands tested similarly affected *in vitro* Th17 function, the endogenous ligand FICZ proved to exert substantial suppression of EAE responsiveness - albeit lower than that by TCDD - if administered systemically by peritoneal injection. This indicates that AhR mediated effects on different cell types are integrated in the final response *in vivo*. AhR signalling seems to be tightly controlled under physiological conditions via induction of the downstream CYP450 family members CYP1A1, CYP1A2 and CYP1B1 that metabolise endogenous ligands, thereby curtailing AhR signalling [20]. Additional feedback controls via induction of the AhRR [46] as well as proteasomic degradation of AhR are in place to control AhR activity [47]. Prolonged signalling occurs following administration of TCDD, which cannot be metabolised, and it was suggested that this might underlie dysregulated responses in the presence of TCDD [48], [49].

Given that AhR is expressed widely in the immune system and also on epithelial and stromal cells, that under *in vivo* conditions will interact and influence immune cells, we have attempted to restrict responsiveness to AhR by analysing mice with cell type specific deletion of AhR for their susceptibility to EAE. Neither the absence of AhR in CD11c expressing antigen-presenting cells, nor in Rag1 expressing immune cells influenced the induction and the course of EAE. However, restricting AhR deficiency to either cell type partly abolished the suppressive effect of TCDD. This suggests that both antigen presenting cells as well as T cells are targets for TCDD, so that suppression of either component will result in a degree of disease amelioration. It is known that immunoregulatory features such as the production of IDO by antigen presenting cells is enhanced by AhR stimulation [50], [51] and the kynurenine pathway also was suggested to influence IL-10 production by dendritic cells [16]. We observed for instance that dendritic cells isolated from draining lymph nodes of mice immunized for induction of EAE together with systemic application of FICZ showed considerably higher expression of IL-10 (**Fig. S1**). Taken together our data suggest that AhR ligands exert complex influences on many cell types, including antigen-presenting cells and T cells, which during EAE are integrated in downstream Th17-mediated immune *sequelae.* Furthermore, time as well as route of administration led to distinct outcomes of Th17 dependent immune responses. More studies are needed to elucidate on the molecular level what the effects of this transcription factor are on different immune cell types (as well as epithelial and stromal cells) that participate in the generation of beneficial as well as pathogenic immune responses *in vivo*.

## Supporting Information

Figure S1 **Dendritic cells from mice immunized simultaneously with systemic AhR stimulation express increased levels of IL-10.** **A**) CD11c+ cells were sorted from either draining lymph nodes (dLN) or spleen (spl) at day 12 after immunization and il10 expression was determined by qRT-PCR. n = 1-6
(PDF)
