# Supplementary figures and images for: Differential Influences of the Aryl Hydrocarbon Receptor on Th17 Mediated Responses in vitro and in vivo
Source: PLoS One. 2013 Nov 14;8(11):e79819. doi: 10.1371/journal.pone.0079819 (PMC3828240; doi:10.1371/journal.pone.0079819)

**A***il10*

dLN

spl

**Relative expression  
(hpert)  $\times 10^3$** 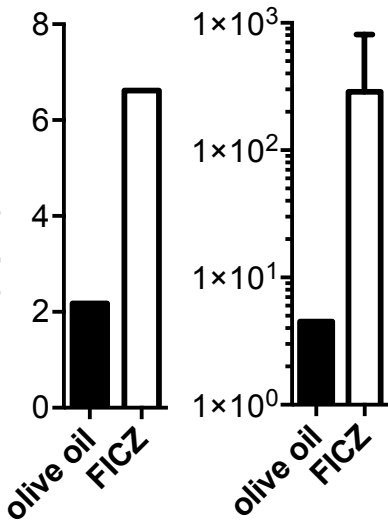

Supplement: Figure S1 — Dendritic cells from mice immunized simultaneously with systemic AhR stimulation express increased levels of IL-10. A) CD11c+ cells were sorted from either draining lymph nodes (dLN) or spleen (spl) at day 12 after immunization and il10 expression was determined by qRT-PCR. n = 1-6 (PDF) [file pone.0079819.s001.pdf]
